# Thioredoxins m regulate plastid glucose-6-phosphate dehydrogenase activity in Arabidopsis roots under salt stress

**DOI:** 10.3389/fpls.2023.1179112

**Published:** 2023-06-02

**Authors:** Guillaume Née, Fuzheng Wang, Gilles Châtel-Innocenti, Amna Mhamdi, Eugénie Juranville, Hélène Vanacker, Graham Noctor, Emmanuelle Issakidis-Bourguet

**Affiliations:** Université Paris-Saclay, CNRS, INRAE, Univ Evry, Institute of Plant Sciences Paris-Saclay (IPS2), Gif sur Yvette, France

**Keywords:** glucose-6-phosphate dehydrogenase, redox regulation, Arabidopsis root, salt stress, plastid thioredoxins

## Abstract

Plants contain several NADPH-producing enzymes including glucose-6-phosphate dehydrogenases (G6PDH) with different sub-cellular localizations. The activity of plastidial G6PDHs is redox-regulated by thioredoxins (TRX). Although specific TRXs are known to regulate chloroplastic isoforms of G6PDH, little information is available for plastidic isoforms found in heterotrophic organs or tissues. Here, we investigated TRX regulation of the two G6PDH plastidic isoforms of Arabidopsis roots during exposure to a mild salt stress. We report that *in vitro* m-type TRXs are the most efficient regulators of the G6PDH2 and G6PDH3 mainly found in Arabidopsis roots. While expression of the corresponding *G6PD* and plastidic *TRX* genes was marginally affected by salt, it impaired root growth of several of the corresponding mutant lines. Using an *in situ* assay for G6PDH, G6PDH2 was found to be the major contributor to salt-induced increases in activity, while data from ROS assays further provide *in vivo* evidence that TRX m acts in redox regulation during salt stress. Taken together, our data suggest that regulation of plastid G6PDH activity by TRX m may be an important player regulating NADPH production in Arabidopsis roots undergoing salt stress.

## Introduction

Plants use NADPH in many metabolic processes, such as assimilation of carbon and nitrogen and biosynthesis of essential compounds (e.g. fatty acids, isoprenoids and aromatic amino acids) for growth and development. NADPH is also a source of reducing equivalents for reactive oxygen species (ROS) scavenging enzymes (Noctor et al., 2015). In plant leaves, during the light period, NADPH is generated primarily through the photosynthetic electron transport chain, but, in darkened leaves and in heterotrophic organs or tissues (e.g. seeds, roots), NADPH is mainly produced from glucose by the oxidative pentose phosphate pathway (OPPP), as in animals and prokaryotes. The OPPP also plays multiple and essential roles in the primary metabolism of plant cells (Kruger and von Schaewen, 2003). In root heterotrophic plastids, the OPPP supports nitrite reduction for nitrate assimilation (Bowsher et al., 1989; Esposito et al., 2001; Esposito et al., 2005; Bussell et al., 2013). Glucose-6-phosphate dehydrogenase (G6PDH, EC 1.1.1.49) catalyses the first and rate-limiting step of the OPPP, one of two NADPH-generating steps in the pathway (Kruger and von Schaewen, 2003; and references therein). A large number of studies have revealed that G6PDH enzymes are important for an optimal plant growth and development, especially under challenging conditions (Recently reviewed by Esposito, 2016; Jiang et al., 2022).

In plant cells, G6PDH isoforms are found in the cytosol, chloroplasts and peroxisomes. In the model plant Arabidopsis, the *G6PD* gene family comprises six members (Wakao and Benning, 2005). Two genes (*G6PD5* and *G6PD6*) encode cytosolic enzymes. The other *G6PD* genes code for proteins with an N-terminal chloroplast targeting peptide but only three of them (G6PDH1, G6PDH2 and G6PDH3) are functional (Wakao and Benning, 2005; Meyer et al., 2011). Among plastid isoforms, G6PDH1 is the most abundant in chloroplasts, and can be alternatively targeted to peroxisomes *via* its cysteine-dependent interaction with G6PDH4 in the cytosol (Meyer et al., 2011). G6PDH2 and G6PDH3 are the isoforms mostly found in heterotrophic plastids, including in roots (Wakao and Benning, 2005).

While the NADPH to NADP^+^ ratio is considered as a possible mechanism for regulation of the activity of the cytosolic isoforms (Cardi et al., 2015), plastid G6PDHs are mainly regulated by a post-translational redox mechanism that allows the OPPP to respond to changes in light conditions. In the chloroplast, G6PDH activity is reversibly inhibited by light to allow efficient photosynthesis through avoidance of futile cycling of the reversible enzymatic steps shared by the OPPP and the Calvin-Benson-Bassham cycle. In the dark, this inhibition is alleviated and the OPPP provides reducing equivalents in the form of NADPH. This key redox- tuning process occurs through the regulation of G6PDH activity by the action of thioredoxins (TRXs) (Esposito, 2016, and references therein).

TRXs are small ubiquitous thiol oxidoreductases with a large number of important cellular functions. Plant TRX display a notable high diversity, and functional data obtained *in vitro* together with recent *in planta* studies revealed that plastidial isoforms can have specific functions (Reviewed by Geigenberger et al., 2017; and references therein). In the model plant Arabidopsis, half of the 20 canonical TRX isoforms present are plastid-localized isoforms and, based on their sequence homologies, they were divided into five sub-types: the f, m, x, y and z types (Meyer et al., 2012). Initial biochemical studies showed that some plastidial TRX isoforms are more efficient as regulators of enzyme activities while others act as reducing substrates for enzymes such as peroxidases that function to remove ROS. Generally, the regulation of carbon metabolism is attributed to the f and m-types, the x and the y-type isoforms have antioxidant functions, and the role of TRX z seems to be confined to the regulation of plastid gene expression (Geigenberger et al., 2017). This overall functional specialization has been confirmed *in planta*, but so far studies have mostly been conducted on leaves, with little information available for other organs such as roots. In terms of their importance in environmental responses, some chloroplast TRXs are important for adapting photosynthesis to fluctuating light conditions (Nikkanen and Rintamäki, 2014; Thormählen et al., 2017), and others for tolerance to abiotic stresses such as high light or drought (Laugier et al., 2013; Vanacker et al., 2018).

We previously found that G6PDH1 is oxidatively activated and reductively inhibited by TRXs (Née et al., 2009). By contrast, neither H_2_O_2_ nor the glutathione/glutaredoxin system seem to be directly involved in the redox regulation of this enzyme (Née et al., 2009). Hence, regulation of plastid G6PDH requires the intervention of TRX. Previous studies also suggest that TRXs modulate the activity of the various G6PDH plastidial isoforms with different efficiencies. We found that Arabidopsis TRX f1, TRX m1 and TRX m4 are equivalent efficient regulators (activators and inhibitors) of G6PDH1 (Née et al., 2009). Notably, we also found that TRX y1, while being as efficient as TRXs f and m for G6PDH1 activation, is a much less efficient inhibitor of the enzyme (Née et al., 2009). These findings illustrate the interest of testing TRX regulatory capacities of all plastidial TRX types towards G6PDH and in both reducing and oxidizing conditions to reveal their functional specificities. TRX f was proposed to be a poor regulator of heterotrophic plastid G6PDH isoforms from potato and poplar. Wenderoth et al. (1997) found that a potato plastidic G6PDH was efficiently activated by a spinach TRX m, and not by a spinach TRX f. Cardi et al. (2016) tested the reductive deactivation of one of the two poplar G6PDH plastidic isoforms, and found that spinach TRX f was less effective than spinach TRX m, and that poplar TRX z was totally inefficient. Based on these findings, G6PDH isoforms might be regulated by TRXs m in non-photosynthetic plastids. However, there has been no comprehensive examination of the ability of the different TRX types to regulate plastid G6PDH activities in roots.

In the present work, we exhaustively tested the capacity of Arabidopsis TRXs of the various plastidial types to regulate (activate or inhibit) the activity of the two homologous G6PDH isoforms preferentially expressed in non-photosynthetic plastids of Arabidopsis. We show that m-type TRXs are the best regulators of G6PDH2 and G6PDH3 *in vitro*, and that their deficiency consistently decreases G6PDH activity in Arabidopsis roots tips. We also provide evidence that TRXs m are important for root growth and tolerance to salinity.

## Materials and methods

### Plant materials

All Arabidopsis mutants used are in the Columbia (Col-0) ecotype except the *trxm3* mutant obtained in the Landsberg (Ler) ecotype. The *g6pd* single mutants, *g6pd2* (SAIL_1240_G11), *g6pd3* (SALK_139479), *g6pd5* (SAIL_97_F06) and *g6pd6* (SALK_016157C) mutants were already genetically described (Wakao and Benning, 2005; Yang et al., 2019). Because *G6PD5* and *G6PD6* encoding cytosolic isoforms were already showed to be functionally redundant (Yang et al., 2019), the corresponding mutants were manually crossed to generate a double mutant (*g6pd5.g6pd6*: *g6pd5.6*). The genetic details of the *trx* mutants were previously described. The *trx* mutants with single or multiple mutations used in this work were*: trxf1.trxf2* (*trxf1.f2*) (SALK_128365/SALK_123826) (Thormählen et al., 2013; Yoshida et al., 2015); *trxy1.trxy2* (*trxy1.y2*) (SALK_103134/SALK_028065) (Née et al., 2021); *trxm1.trxm2.trxm4* (*trxm1.2.3*) (WiscLox375F05/SALK_130686/SALK_023810) (Laugier et al., 2013; Okegawa and Motohashi, 2015; Thormählen et al., 2017); *trxm3* (ET3878) (Benitez-Alfonso et al., 2009); *trx x* (SALK_128906) (Pulido et al., 2010); and *trx z* (SALK_059334C) (Arsova et al., 2010).

### Growth conditions

Arabidopsis (*Arabidopsis thaliana*) wild-type (Columbia: Col-0, or Landsberg: Ler ecotype) and mutant (detailed above) seeds were surface sterilized and sown on agar (0.6% w/vol) containing standard half Murashige and Skoog medium (½MS). For salt stress conditions, the agar medium was supplemented with 100 mM NaCl, causing a mild salt stress. Germination was synchronized by a cold treatment at 4°C for 72 hours. Short-day growth conditions were used (growth chamber): 8-hr day photoperiod, 80-100 µmol/m^2^/s, 20°C. For experiments, seedlings were carefully removed from the slurry agar medium (with or without NaCl) for analyses. For root growth monitoring, plants were positioned horizontally on the surface of solid agar medium and the length of primary roots was measured. For *in situ* G6PDH activity assays (NBT reduction) and ROS measurements (H2-DCFDA probe) roots were further incubated in chemical reagents, as described below.

### Recombinant proteins

G6PDH2 and G6PDH3 isoforms were produced in *E. coli* using the strep-tag technology (C-terminally tagged mature proteins) as previously described (Wakao and Benning, 2005). TRX proteins (mature forms) were recombinantly obtained as we previously detailed (Collin et al., 2003; 2004; Bohrer et al., 2012). G6PDH and TRX proteins used in this study were purified to homogeneity (appearing as single protein bands of ca. 60 kDa and 12 kDa, respectively, after SDS-PAGE and Coomassie staining). To improve protein stability upon storage, NADP^+^ was added to G6PDH protein preparations (Wakao and Benning, 2005).

### Gene expression level analyses

Total RNA was isolated from 30-100 mg of frozen plant leaves and roots as described in Bohrer et al. (2012). Gene expression profiling by real-time quantitative RT-PCR was performed in triplicate reactions for each sample and using gene-specific primer pairs (detailed in Bohrer et al. (2012) for *TRX* genes and in **Table S1** for *G6PD* genes), as previously described (Bohrer et al., 2012). The results were standardized to the *PP2A* reference gene whose expression remains constant in different organs and in stress conditions (Czechowski et al., 2005).

### *In situ* G6PDH activity assay in Arabidopsis roots

8 day-old seedlings were fixed and permeabilized in 7.5% (w/v) paraformaldehyde in MTSB (MicroTubules Stabilization Buffer): 50 mM PIPES, 5 mM EGTA, 5 mM MgSO_4_, pH 6.9, at 4°C for 1 h; then rinsed extensively in HEPES 100 mM pH 7.4 at 4°C (5 times for 10 min) to remove soluble carbohydrates. Staining for G6PDH activity was done by incubating seedlings for 10 min at 30°C in the enzyme assay mixture containing: 10 mM glucose-6-phosphate, 1 mM NADP^+^, 5 mM MgCl_2_, 0.7% (w/vol) BSA, protease inhibitor cocktail (special plant, SIGMA) and 0.05% NBT (w/v). Finally, roots were rinsed in HEPES buffer and mounted between a slide and a coverslip for observation using a multizoom microscope (AZ 100 NIKON) equipped with a camera. Images were captured and treated using the NIS-Elements BR software. The intensity of staining in root tips was quantified using Image J (Schneider et al., 2012) and expressed as a percentage of coloration, where the intensity of coloration of the wild-type was set to 100%.

### Root length measurement

Experiments with the various genotypes were performed using at least two different seed batches. Mutants were always compared to wild-type of the corresponding ecotype using seeds from parent plants grown in the same time and in the same conditions. Arabidopsis seedling images were scanned at the resolutions of 600–800 dots per inch. Primary root lengths were measured using ImageJ (Schneider et al., 2012).

### Imaging of H_2_O_2_ in Arabidopsis root tips using the fluorescent probe H2-DCFDA

Imaging was performed using a fluorescence microscope (ZEISS Axiolmager Z2) fitted with a GFP filter.

### Reagents

All chemical reagents used were from Sigma-Aldrich or LifeTechnologies, except the reagents for real-time quantitative PCR obtained from Roche Applied System.

### Statistical analysis

Two-tailed Student’s t-tests were used for two-group comparisons (mutant *vs.* wild-type for a given growth condition, or salinity *vs.* control for a given genotype).

## Results

### Arabidopsis plastid G6PDH isoforms are most efficiently regulated by m-type TRX *in vitro*

**Figure 1:**
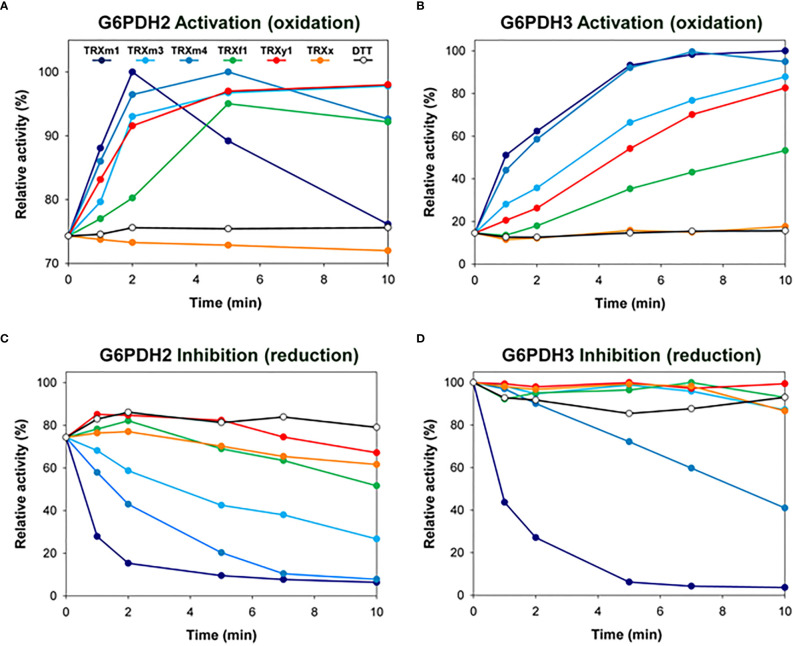
Redox regulation of G6PDH activity by various TRX *in vitro.* Redox regulation of G6PDH2 and G6PDH3 was tested by incubating the enzyme with **(A, B)** oxidized (in presence of 10mM DTTox) or **(C, D)** reduced (in presence of 1 mM DTTred) TRX (at a concentration of 10µM), prior to enzyme activity assays. DTT alone (o) had no effect at working concentrations. G6PDH2 and G6PDH3 showing an initial activity corresponding respectively to 75% and 15% of their maximal activity, activation kinetics (performed without a pre-reduction treatment) are shown with different ranges in the Y-axes in panels **(A, B)**. Experiments were reproduced at least 2 times using independent recombinant G6PDH preparations. Representative curves are shown.

G6PDH2 and G6PDH3 proteins were recombinantly produced in their mature form and purified by affinity chromatography (strep-tag added at the C-terminus) (Wakao and Benning, 2005). They showed a substantial initial G6PDH activity and were both sensitive to redox treatments. After full activation by oxidation, G6PDH2 and G6PDH3 reached comparable activities of 39.6 and 46.9 U/mg, respectively. Although they were obtained from *E. coli* following the same procedure, G6PDH2 and G6PDH3 differed in their initial redox state, respectively corresponding to 75% and 15% of their maximal activity (measured after oxidation) (**Figures 1A, B** **)**. When we tested and compared various plastid TRX proteins of the different types for their capacity to redox-regulate G6PDH activity, we found that both G6PDH2 and G6PDH3 are efficiently activated by TRX m1, m3 and m4 (**Figures 1A, B** **)**. In reducing conditions, TRX m1 and m4 were the most effective inhibitors of plastid G6PDH activity (**Figures 1C, D** **)**, while TRXm3 was less potent towards G6PDH2 (**Figure 1C**) and totally inefficient with G6PDH3 (**Figure 1D**). TRX f1 proved to be a poor activator (**Figures 1A, B** **)** and an inefficient inhibitor (**Figures 1C, D** **)** of both G6PDH2 and G6PDH3. As previously found with G6PDH1 (Née et al., 2009), TRX x does not efficiently regulate the activity of other plastid G6PDHs (**Figure 1**), and TRX y1 was ineffective in reduction (**Figures 1C, D** **)**, although it showed a good or moderate capacity to activate G6PDH2 and G6PDH3, respectively (**Figures 1A, B** **)**. Overall, these data obtained *in vitro* using recombinant proteins suggest that m-type TRXs are the most efficient regulators of the activity of plastid G6PDH isoforms occurring in non-photosynthetic tissues.

Next, we investigated whether these functional specificities found *in vitro* for the different plastid TRX isoforms could have a physiological significance *in planta*. In Arabidopsis, the two genes encoding heterotrophic plastid G6PDH isoforms are mainly expressed in roots (Wakao and Benning, 2005), and in this organ *TRXM*-encoding genes are substantially expressed, together with *TRXX* and *TRXY1* (Bohrer et al., 2012). In the present work, we specifically addressed the role of heterotrophic plastid G6PDH and TRX isoforms in Arabidopsis roots. To avoid illumination of roots, which can induce ROS production (Yokawa et al., 2011) and affect root morphogenesis (Xu et al., 2013), we set up an experimental system in which Arabidopsis seedlings were grown vertically in a box allowing shoots exposure to the light while root grew in the dark (**Figure S1A**). This system allowed us to circumvent light effects, and to specifically study physiologically relevant functions of G6PDH and TRX in the roots.

### G6PDH2 and G6PDH5/6 mainly contribute to Arabidopsis root growth and salt tolerance

**Figure 2:**
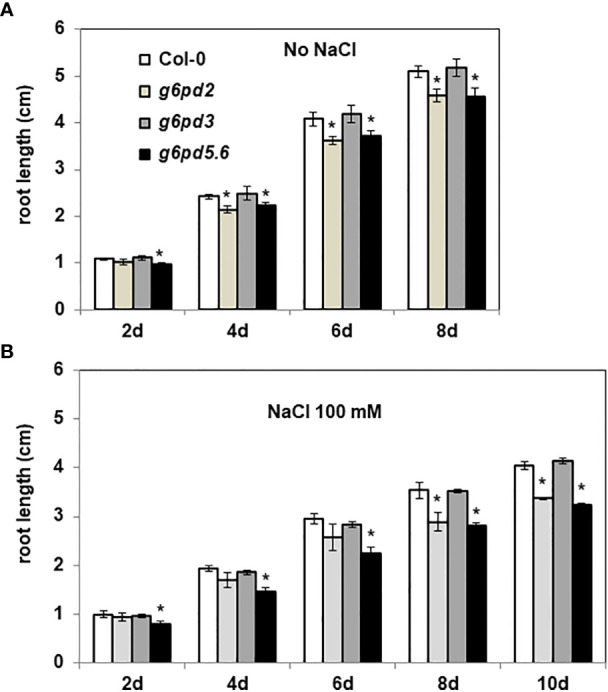
Root length of *g6pd* mutants. 4 day-old seedlings germinated on ½ MS medium were transferred onto new media **(A)** without or **(B)** with salt (100 mM NaCl). Root lengths were measured every 2 days. Bars indicate standard deviation. * indicate a value significantly different from Col-0, with *P* < 0.05, according to Student’s *t* test (n = 20).

We first evaluated the importance of G6PDH isoforms for root growth. In control conditions, after one week from sowing, we observed that both *g6pd2* and *g6pd5. g6pd6* (*g6pd5.6*) mutants had shorter roots than Col-0 plants (**Figure S1B**). By monitoring root growth from 2 days to 8 days after sowing, we found that, in control conditions, the roots of *g6pd2* and *g6pd5.6* mutants grew significantly more slowly compared with the wild-type (Col-0 ecotype) and the *g6pd3* mutant (**Figure 2A**). In the presence of a mild salt stress (100 mM NaCl), differences between genotypes were even more pronounced (**Figure 2B**), especially after 8 days when the root growth of the *g6pd2* and *g6pd5. 6* mutants were impacted by *ca.* 40%. These results suggested a possible role of *G6PD2* and *G6PD5* and/or *G6PD6* genes in root growth and in salt tolerance of Arabidopsis.

### TRXs of the m subtype contribute to Arabidopsis root growth and salt tolerance

**Figure 3:**
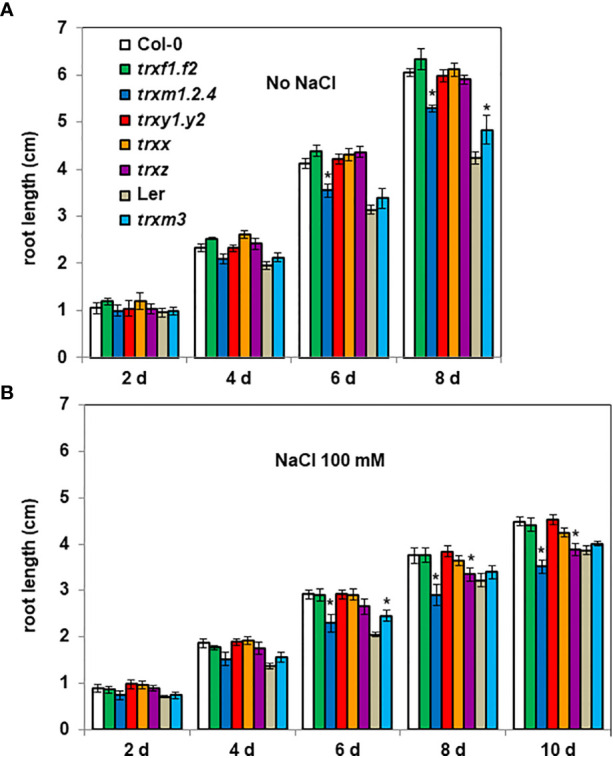
Root length of *trx* mutants. 4 day-old seedlings germinated on ½ MS medium were transferred onto new media **(A)** without or **(B)** with salt (100 mM NaCl). Root lengths were measured every 2 days. Bars indicate standard deviation. * indicate a value significantly different from WT (Col-0 or Ler), with *P* < 0.05, according to Student’s *t* test (n = 20).

We also monitored the root growth of *trx* mutants. In the absence of stress, compared with Col-0 seedlings, the *trxm1. trxm2. trxm4* (*trxm1.2.4*) triple mutant had shorter roots from 6 days after seed sowing (after 8 days, *trxm1.2.4* showing *ca.* -10% length of the primary root) (**Figure 3A**). By contrast, the *trxm3* mutant had longer roots compared with Ler seedlings (after 8 days, *trxm3* showing *ca.* +10% length of the primary root) (**Figure 3A**).

By monitoring darkened root growth we observed that Col-0 had longer roots compared with Ler (**Figure 3A**), the difference between the two Arabidopsis ecotypes being less marked in presence of NaCl (Col-0 roots *ca.* 15% longer than Ler roots) compared to the control condition (Col-0 roots *ca.* 40% longer than Ler roots) (**Figure 3B**). At 8 d, 100 mM NaCl in the medium similarly affected the root growth of both ecotypes (*ca.* - 38% and - 34% length of the primary root, respectively for Col-0 and Ler ecotypes), but at a higher salt concentration (200 mM NaCl), Ler showed a clearly higher sensitivity than Col-0 to salinity (**Figure S2**).

When we tested the possible contribution of the various TRX isoforms to salt tolerance, we found that, in the presence of 100 mM NaCl and in contrast to their corresponding wild-type (WT), the *trxm3* mutant had (as in the absence of salt) a better root growth capacity (after 8 days, *trxm3* showing *ca.* + 15% length of the primary root), while the root growth of the *trxm1.2.4* and the *trxz* mutants were more severely affected by salt stress (**Figure 3B**). After 8 days, salinity decreased the Col-0 root growth by 38%, while the *trxm1.2.4* and the *trxz* mutants were significantly more impacted, by 45% and 43%, respectively. These mutant phenotypes were even more apparent at a higher salt concentration (**Figure S2**). Taken together, these data suggest that TRXs m, together with TRX z, are important for Arabidopsis root growth and salt tolerance.

### The various G6PDH and TRX isoforms are differentially expressed in Arabidopsis roots

Next, we wondered whether the mutant root growth phenotypes we observed could reflect differences in gene expression levels. First, we analyzed and compared transcript levels of the six Arabidopsis *G6PD* genes, in the leaves and in the roots of Arabidopsis plants cultivated in our growing conditions. We found that *G6PD5* was comparably strongly expressed in both organs, and that, among the genes encoding plastid isoforms, *G6PD1* and *G6PD3* were the most highly expressed in leaves and roots, respectively (**Figure S3A**). We also detected a substantial expression of *G6PD2* and *G6PD6* genes in the roots, as previously reported (Wakao and Benning, 2005). We have reported elsewhere that the genes encoding m-type TRXs, TRX x and TRX y1 isoforms are substantially expressed in Arabidopsis roots, while the expression of f-type TRX isoforms is restricted to green tissues/organs (Bohrer et al., 2012).

Overall, the expression analyses of *G6PD* and *TRX* genes suggest that some of the corresponding plastid isoforms are present in Arabidopsis roots and can be redox partners.

### The effect of salt stress on transcript levels of *G6PD* and *TRX* genes is weak

**Figure 4:**
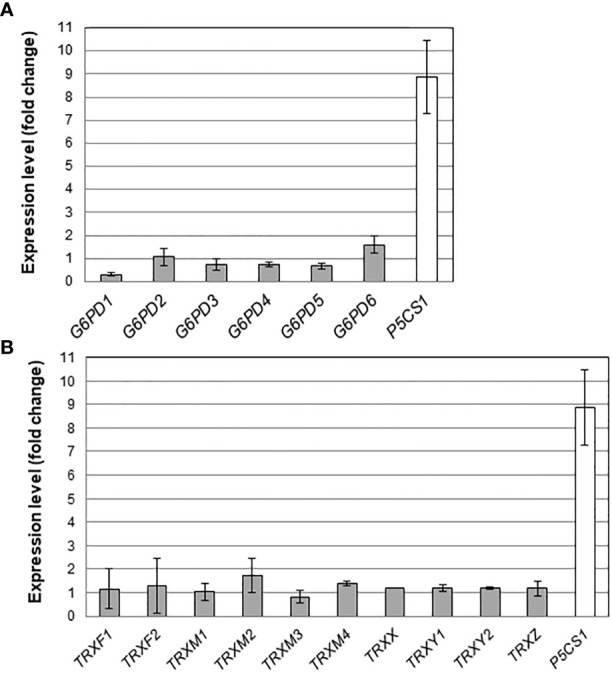
Effect of salt stress on root mRNA levels. Transcripts for **(A)** G6PDH and **(B)** plastidial TRX isoforms were quantified in Arabidopsis roots by quantitative RT-PCR. For comparison, the stress responsive gene *P5CS1* (Funck et al., 2020) was also analysed. Bars indicate standard deviation (n = 6).

We also measured transcript levels of *G6PD* and *TRX* genes in the roots of plants growing under a mild salt stress. As a positive control for the effect of NaCl on gene expression, we also measured mRNA transcripts of the *P5CS1* gene, a gene activated by salt and responsible for proline accumulation in Arabidopsis (Funck et al., 2020). In the roots of Col-0 plants, in our growing conditions, while *P5CS1* was strongly induced (about 10 times) in presence of 100 mM NaCl, the expression levels of *G6PD* genes were not significantly impacted (**Figure 4A**). We also found that salt stress had a marginal effect on the expression levels of plastid TRX-encoding genes (**Figure 4B**).

Overall, the effect of salt on transcript levels of *G6PD* and *TRX* genes in darkened roots was weak.

### G6PDH activity is strongly induced by salt stress in Arabidopsis root tips

To measure G6PDH activity in the roots of Arabidopsis, we implemented an *in situ* colorimetric assay. In this assay, the G6P-dependent production of NADPH is monitored by the reduction of NBT, reduced NBT forming a visible formazan that precipitates *in situ* (**Figure S4A**). We observed a strong coloration in the transition zone of root tips, between the distal meristem zone and the elongation zone. In control experiments (**Figures S4B**), staining was much lower (without G6P) or totally absent (without NADP^+^) in this area of the root tip, validating the specificity of the signal for measuring *in situ* G6PDH activity. In the roots of WT seedlings grown in the presence of 100 mM NaCl, we found a marked increase of the NBT staining signal. Thus, despite the weak responses to salt at the transcript level (**Figure 4A**), root G6PDH activity was substantially induced by salt stress (**Figure S4C**).

### G6PDH2 is the main contributor to the induction of G6PDH activity by salt stress in root tips

In WT seedlings, we could estimate a two-fold induction of G6PDH activity in the root tips under salt stress (factor of 2.1 and 1.8 in Col-0 and Ler Arabidopsis ecotypes, respectively).

**Figure 5:**
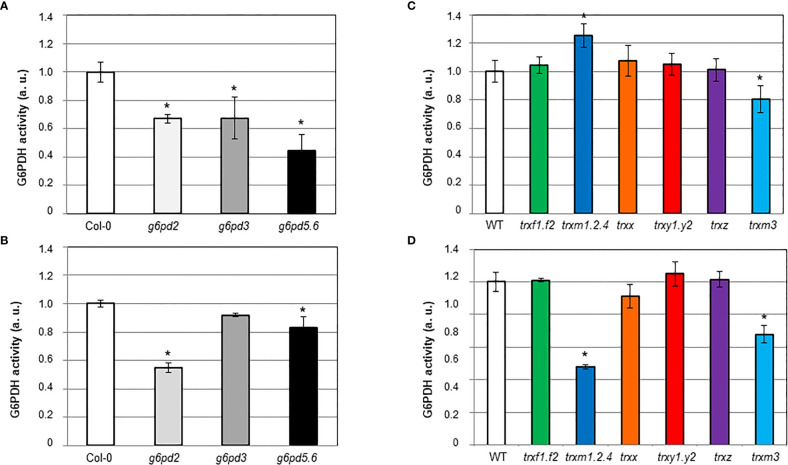
*In situ* G6PDH activity in Arabidopsis root tips of *g6pd* and *trx* mutants. G6PDH activity was measured on 8-d old seedlings: **(A, C)** in control condition; **(B, D)** in the presence of 100 mM NaCl. At least 3 independent experiments were performed for each genotype grown in each condition, by an implement staining method as detailed in the Materials and methods section, and exemplified in **Figure S4**. In each set of analyses data were normalized to WT grown in control condition. Bars indicate standard deviation. *indicate a value significantly different from WT with *P*<0.05, according to Student’s *t* test (n = 9-12).

In control conditions, the G6PDH activity was significantly decreased in all *g6pd* mutants compared to Col-0 (**Figure 5A**). The induction of G6PDH activity by salt stress was comparable in Col-0 and in the *g6pd3* mutant, while it was significantly lower in *g6pd5.6* and nearly annulled in *g6pd2* (**Figure 5B**).

These results suggest that plastid and cytosolic G6PDH isoforms contribute to root enzyme activity in control conditions, and reveal that G6PDH2 is the predominant contributor to the increase in activity in response to salt stress.

### Loss of m-type TRX function specifically impairs salt-induction of G6PDH activity in root tips

To establish whether TRX isoforms were involved in the induction of G6PDH by salt, we measured G6PDH activity in the root tips of the *trx* mutants (**Figures 5C, D** **)**. Mutations in m-type TRXs especially impacted G6PDH activity. In the triple *trxm1.2.4* mutant, the enzyme activity was markedly increased (*ca.* 25% more than Col-0) in control conditions (**Figure 5C**), and decreased (*ca.* 52% less than Col-0) under salt stress (**Figure 5D**). In the *trxm3* single mutant, G6PDH activity was decreased both in control and salt stress conditions (*ca.* 19% and 32% less than Ler, respectively). Deficiency of other TRX isoforms did not significantly affect root G6PDH activity.

Taken together, these results suggest that m-type TRXs are important to modulate G6PDH activity in Arabidopsis root tips, especially under salt stress conditions.

### G6PDH2 plays a major antioxidant role in the roots growing under salinity

**Figure 6:**
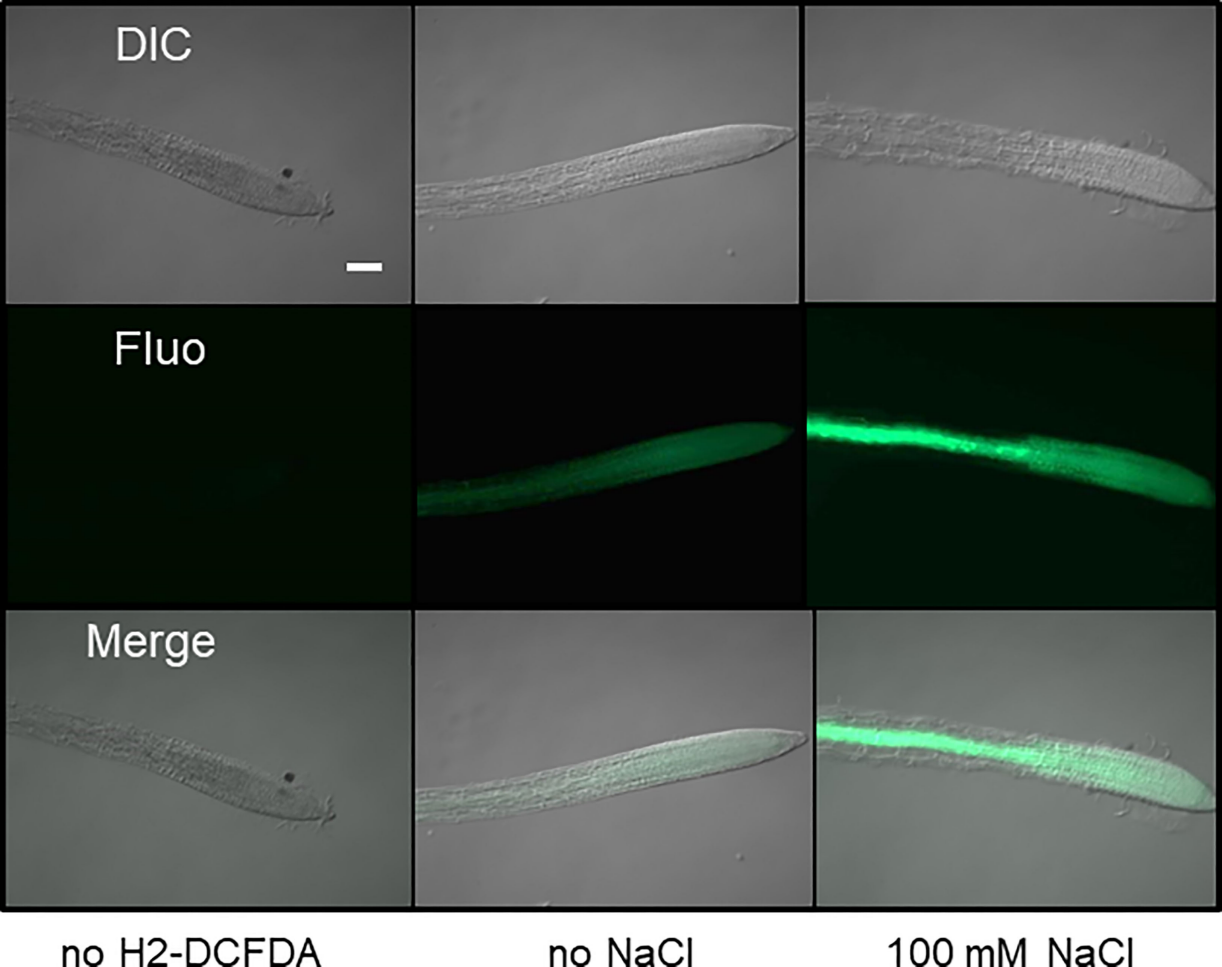
Imaging of ROS in Arabidopsis root tips. Roots from 1-week old seedlings were stained with the fluorescent probe H2-DCFDA to reveal H_2_O_2_ in root tips. Representative images (DIC:differential interference contrast; Fluo: fluorescence) are shown for control (without probe), and roots grown in absence or presence of salt. Bar = 10µm. Exposure time 300 ms.

**Figure 7:**
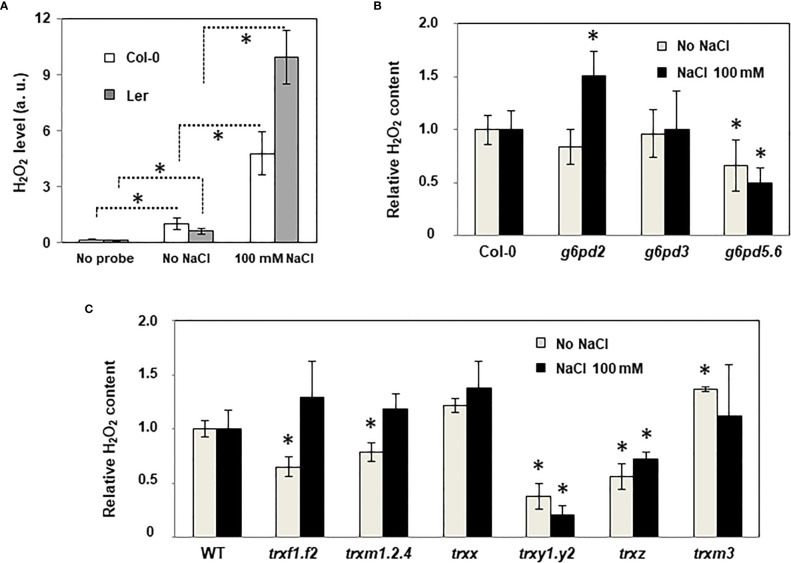
ROS levels in the roots of WT and mutant plants under salt stress. Quantification of ROS in root tips using H2-DCFDA fluorescence probe. **(A)** Effect of salt stress in WT ecotypes (Col: Columbia, Ler: Landsberg); **(B)** Effect of salt stress in *g6pd* mutants; **(C)** Effect of salt stress in *trx* mutants. Pixel intensity in intact root tips was quantified using the Image J software (as described in **Figure S4**). Data are means from three experimental replicates with at least 4 root tips analysed per sample. Bars indicate standard deviation. In **(B, C)**, ROS relative content is expressed as the percentage relative to WT seedlings (Col-0 ecotype for all mutants except for *trxm3* in Ler ecotype). * indicate significantly different values in **(A)**, values significantly different from WT in **(B, C)**; with *P*<0.05, according to Student’s *t* test (n = 12-18).

Because the antioxidant function of G6PDHs was well known (Izawa et al., 1998; Jin et al., 2021), we estimated ROS levels in Arabidopsis root tips using the H2-DCFDA fluorescent probe. In WT plants, salt stress provoked a strong increase of the fluorescent signal, especially in the vasculature (**Figure 6**). In the root tip, in the area of the elongation zone (where G6PDH activity was mainly detected, **Figures S4B, C**), the fluorescent signal was enhanced by a factor of 4.8 and 9.9, in Col-0 and Ler ecotypes, respectively (**Figure 7A**). Compared with Col-0, while the *g6pd3* mutant exhibited comparable ROS levels, the *g6pd5.6* mutant showed decreased ROS signals in both control and salt conditions, and *g6pd2* showed an increased root ROS content in presence of NaCl (**Figure 7B**).

These data suggest that G6PDH2 plays a major antioxidant role in the root growing under salinity.

### TRX deficiencies differently impact ROS levels in the root

Since the mutation of *TRX* genes may primarily impact the redox status of root cells and, consequently, redox regulation of G6PDH activity, we also measured ROS levels in the root tips of *trx* mutants (**Figure 7C**). In control conditions, we found that ROS levels were impacted by all *TRX* gene mutations, with the exception of *TRXx*. Levels were significantly decreased in *trxf1.f2*, *trxm1.2.4* and *trxz* mutants, and most markedly in the *trxy1.y2* mutant, while it was increased in *trxm3*. Under salt stress, compared with WT, the *trxy1.y2* and *trxz* mutants showed a substantially attenuated ROS induction, while it was not significantly different in the other *trx* mutants, including the mutants of the TRX m type.

Taken together, these results reveal a connexion between most plastid TRX and ROS levels in Arabidopsis primary roots, and a specific role is suggested for TRXs of the y and z types in salt stress conditions.

## Discussion

Past studies on G6PDH from several plant species support the idea that, despite some distinct biochemical features, the activity of plastid isoforms from autotrophic or heterotrophic tissues is regulated by thioredoxins (Wendt et al., 1999; Wendt et al., 2000; Esposito et al., 2001; Wakao and Benning, 2005; Cardi et al., 2013; Cardi et al., 2016). In a previous study, we found that TRX f regulates G6PDH1, the most abundant plastid G6PDH isoform in Arabidopsis leaves, as efficiently as TRXs m (Née et al., 2009). Here, we show that TRX f is a less efficient regulator towards G6PDH2 and G6PDH3, the isoforms mainly found in Arabidopsis heterotrophic plastids. Instead, we found that TRXs of the type m, especially TRXm1, are the most efficient regulators of these isoforms (**Figure 1**). This finding is in accordance with past results obtained with potato or poplar G6PDH enzymes tested *in vitro* (Wendt et al., 2000; Cardi et al., 2016). In addition, based on our knowledge about TRX functional redundancies, we used single or multiple mutant lines deficient for specific TRX types to investigate the physiological relevance of plastid G6PDH regulation by TRXs m in the roots under a mild salt stress. We also used reverse genetics to discriminate between plastid and cytosolic G6PDH isoforms and to estimate their relative contributions to G6PDH activity in Arabidopsis roots.

Previous *in planta* studies on G6PDH, mainly focused on cytosolic isoforms and expression levels of their corresponding genes, evidencing the importance of G6PDH for plant tolerance to salt stress in various plant species (in tobacco: Scharte et al., 2009, in wheat: Nemoto and Sasakuma, 2000; in soybean: Zhao et al., 2020; in sugarcane: Yang et al., 2014; in barley: Cardi et al., 2015), including Arabidopsis (Yang et al., 2019; Jin et al., 2021). In barley roots, a substantial and salinity-specific increase in total G6PDH activity was found, and cytosolic G6PDH together with plastid isoforms were proposed to play different roles in the salt stress response (Cardi et al., 2015). In Arabidopsis, over-expression of a cytosolic G6PDH promotes seed germination and root development under salt stress (Jin et al., 2021). Here, we provide evidence that plastid G6PDHs also significantly contribute to root growth and salt tolerance, and we experimentally addressed the functional importance of TRXs m in influencing these physiological traits.

In the control condition (**Figure 5A**), we found a shared contribution between cytosolic and plastid isoforms to global G6PDH activity in Arabidopsis root tips. However, under salt stress, the plastid G6PDH2 isoform plays a predominant role, over cytosolic and G6PDH3 isoforms which show a more limited or no contribution, respectively (**Figure 5B**). We found that m-type TRXs deficiencies significantly impact global G6PDH activity in Arabidopsis root tips. Among all *trx* mutants, *trxm1.2.4* was the only mutant exhibiting a marked increase in G6PDH activity in the control (presumably reducing) condition (**Figure 5C**), together with a lower enzyme activity under salt stress (oxidizing condition) (**Figure 5D**). This finding is in accordance with biochemical data showing that, at the exception of TRXm3, m-type TRX isoforms redox regulate the activity of heterotrophic plastid G6PDHs with a higher efficiency compared to other TRX types. The *trxm3* mutant exhibited unique features, with a constitutively decreased root G6PDH activity (**Figures 5C, D** **)**, and increased ROS levels in the control condition (**Figure 7C**). This finding is in accordance with the increase in hydrogen peroxide content reported in *trxm3* seedlings, suggesting that its root phenotypes may be linked to the function of TRXm3 as a regulator of meristem maintenance (Benitez-Alfonso et al., 2009).

In darkened roots, we found that salt has no effect on either *G6PD* or *TRX* gene expression levels (**Figure 4**) contrasting with previous results showing an induction of plastid G6PDH expression in shoots or leaves during salt stress (Yang et al., 2019; Zhao et al., 2020). In barley, G6PDH activity increased in roots supplied with nitrogen and was accompanied by a specific increase in the protein amount of heterotrophic plastid isoforms (Esposito et al., 2005). The absence of transcriptional regulation suggests that, under salt stress, the increase in global G6PDH activity would be attributable to a post-translational activation of G6PDH2. This is further supported by the fact that, under salt stress, changes in global G6PDH activity were found in specific *trx* mutants, reflecting the impact of TRX deficiency on the regulation. To check whether such effects could be direct (regulation of G6PDH activity by specific TRXs) or indirect (TRX contributing to maintain cellular redox homeostasis), we measured ROS levels as an indicator of the cellular oxidative situation in root tips. This analysis revealed a strong increase in signal in the root tips of Arabidopsis exposed to 100 mM NaCl, confirming previous findings (Jin et al., 2021), and revealing an oxidative stress situation. In comparison with the WT, we found that the loss of *G6PD2* function had no significant effect on root ROS levels in the control condition, but it provoked a strong increase under salinity (**Figure 7B**). In contrast, cytosolic G6PDH deficiency decreased root ROS levels, in both tested conditions (**Figure 7B**). These findings suggest that in Arabidopsis roots, plastid G6PDH isoforms (mainly G6PDH2) and cytosolic G6PDH isoforms would provide NADPH for different functions, possibly to antioxidant enzymes for ROS detoxification, and to NAD(P)H-dependent oxidases to facilitate ROS accumulation for signalling (Scharte et al., 2009), respectively. Since deficiency in either G6PDH2 or cytosolic G6PDH limits Arabidopsis primary root length (**Figure 2**), our results support the importance of G6PDHs in the maintenance of the cellular redox homeostasis for an optimal growth, especially in stress conditions.

Under oxidative conditions, induction of plastidial G6PDH activity requires the specific action of oxidized TRXs potentiated by ROS. Based on the known antioxidant properties of TRXs y (Collin et al., 2004; Navrot et al., 2006; Laugier et al., 2013; Vanacker et al., 2018), and their capacity to regulate the MDHAR activity in Arabidopsis roots (Marchand et al., 2010), elevated ROS levels might be predicted in the *trxy1.y2* mutant. Instead, we found constitutively decreased ROS levels in the roots of this mutant (**Figure 7C**), with no significant impact on their growth (**Figure 3**). Thus, it is as if ROS levels and root growth were disconnected in the absence of TRXs y. Interestingly, in Arabidopsis roots under salt stress, it was previously shown that ROS action on growth is directly related to ABA (Duan et al., 2013), and ABA was found to be important in the induction of G6PDH to counteract stress (Cardi et al., 2011; Esposito, 2016). Taken together with our recent work providing evidence that TRXs y have ABA-related functions in Arabidopsis seeds (Née et al., 2021), the present data further suggest a specific role for TRXs y in hormone-dependent processes in heterotrophic plastids. We also found ROS levels constitutively decreased in the root tips of the *trxz* mutant (**Figure 7C**) which shows a lower capacity to grow under salt stress, compared to WT (**Figure 3**). This finding did not correlate with a significant change in global G6PDH activity (**Figure 5**), consistently with the inability of TRX z to regulate G6PDH activity *in vitro* (**Figure S6**) (Cardi et al., 2016). TRX z is known to be a redox regulator of plastid gene expression, as a component of the plastid encoded RNA polymerase complex (Arsova et al., 2010; Diaz et al., 2018), and a regulator of chloroplast RNA editing (Wang et al., 2021). In soybean chloroplasts, the RNA editing process in transcripts is enhanced by salt stress and correlates with tolerance to salinity (Rodrigues et al., 2017). Based on these findings, it will be of particular interest to validate that the Arabidopsis *trxz* mutant exhibits the RNA editing defects found in the rice mutant (Wang et al., 2021) and whether such effects correlate with its decreased ROS levels and enhanced sensitivity to salt stress (present study). Under salt stress, the lowered G6PDH activity in the *trxm1.2.4* mutant (**Figure 5D**) did not correlate with an increased ROS level (**Figure 7C**). In the control condition, a decreased ROS level was found in this mutant (**Figure 7C**), while in the *g6pd2* and *g6pd3* mutants no significant increase of ROS was measured (**Figure 7B**). Taken together, the present work and the identification of seventy-two TRX potential targets in Arabidopsis roots (Marchand et al., 2010) suggest that TRXs are important for cell redox homeostasis in the roots where their functions largely remain to be explored.

In an earlier study, we showed that the regulation of G6PDH1 activity by TRX occurs through redox-driven structural changes modifying substrate accessibility and cofactor binding (Née et al., 2014). The primary sequence of plastidial G6PDHs is highly conserved and all Arabidopsis isoforms, except G6PDH4, have strictly the same active site sequence (**Figure S6**). Amino acid sequence conservation and 3-D modelling strongly suggest that all TRX-dependent active G6PDH isoforms are regulated by a conserved molecular mechanism driven by the redox state of the regulatory disulfide (Née et al., 2014; **Figures S7**, **S8**). Nevertheless, G6PDH isoforms from chloroplasts and heterotrophic plastids are regulated by TRX f and TRX m with different efficiencies. In Arabidopsis, where TRXs m are considered to be the most abundant isoforms in chloroplasts (Okegawa & Motohashi, 2015) and in root plastids (Sahrawy et al., 2022), reactivities and quantitative considerations thus lead us to conclude that these TRXs are the principal regulators of G6PDHs. In chloroplasts, TRX f would play a complementary role for coordinating the reductive (Calvin-Benson-Bassham cycle) and the oxidative pentose phosphate pathways.

## Data availability statement

The original contributions presented in the study are included in the article/**Supplementary Material**. Further inquiries can be directed to the corresponding authors.

## Author contributions

EI-B, GNé, GNo and FW designed research. GC-I, EJ, AM, GNé, HV and FW performed research. EI-B, GNé, HV and FW analyzed data. And EI-B, GNé and GNo wrote the paper.

## Supplementary material

Supplementary Table 1 Sequences of the primers (forward and reverse) used for *G6PD* genes expression levels analyses by RT-qPCR.
