# Supplementary material for: Thioredoxins m regulate plastid glucose-6-phosphate dehydrogenase activity in Arabidopsis roots under salt stress
Source: Front Plant Sci. 2023 Jun 2;14:1179112. doi: 10.3389/fpls.2023.1179112 (PMC10274509; doi:10.3389/fpls.2023.1179112)
Supplement: Supplementary Table 1 — Sequences of the primers (forward and reverse) used for G6PD genes expression levels analyses by RT-qPCR. [file DataSheet_1.pdf]

Table S1. Sequences of the primers (forward and reverse) used for *G6PD* genes expression levels analyses by RT-qPCR.

G6PD1-QPCR-F1	TGCGTAGATAAATCCTTGTTACC
G6PD1-QPCR-R1	CTCCGAAATGCTCGATTTAG
G6PD2-QPCR-F1	GGTGACGTCAGTATTGACCAG
G6PD2-QPCR-R1	TGGTACATAACCAAATTGCAC
G6PD3-QPCR-F1	GGAAACTTTCTTTCGCTTTCAG
G6PD3-QPCR-R1	AAAGGGTGGCAAGAATAGGG
G6PD4-QPCR-F1	TGGGCAGATGACTGAAGAAG
G6PD4-QPCR-R1	CAGGGAATGGCACAGCTTC
G6PD5-QPCR-F1	TCGGTCCCATACAAACAAGG
G6PD5-QPCR-R1	GGATCCAAATGTAGCCATGAG
G6PD6-QPCR-F1	TCAGACTCACGGCTATATCTGG
G6PD6-QPCR-R1	CGAGGAGGGACTATTCAGCA

(A)

(B)

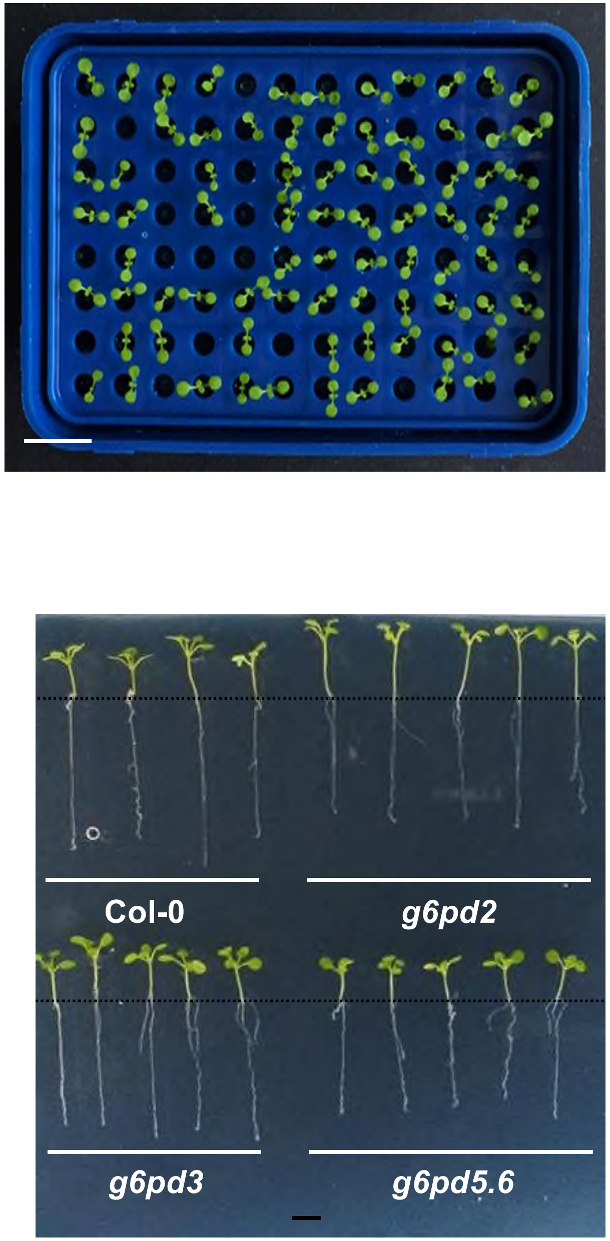

Sup. Figure 1 . Growth system of darkened roots and growth monitoring.

(A) Picture of 4 day-old Col-0 seedlings cultivated in a pipet tip box containing slurry agar ½ MS medium. (B) Root length of 7 day-old Col-0 and *g6pd* mutant seedlings. Scale bar = 1 cm. Representative pictures are shown.

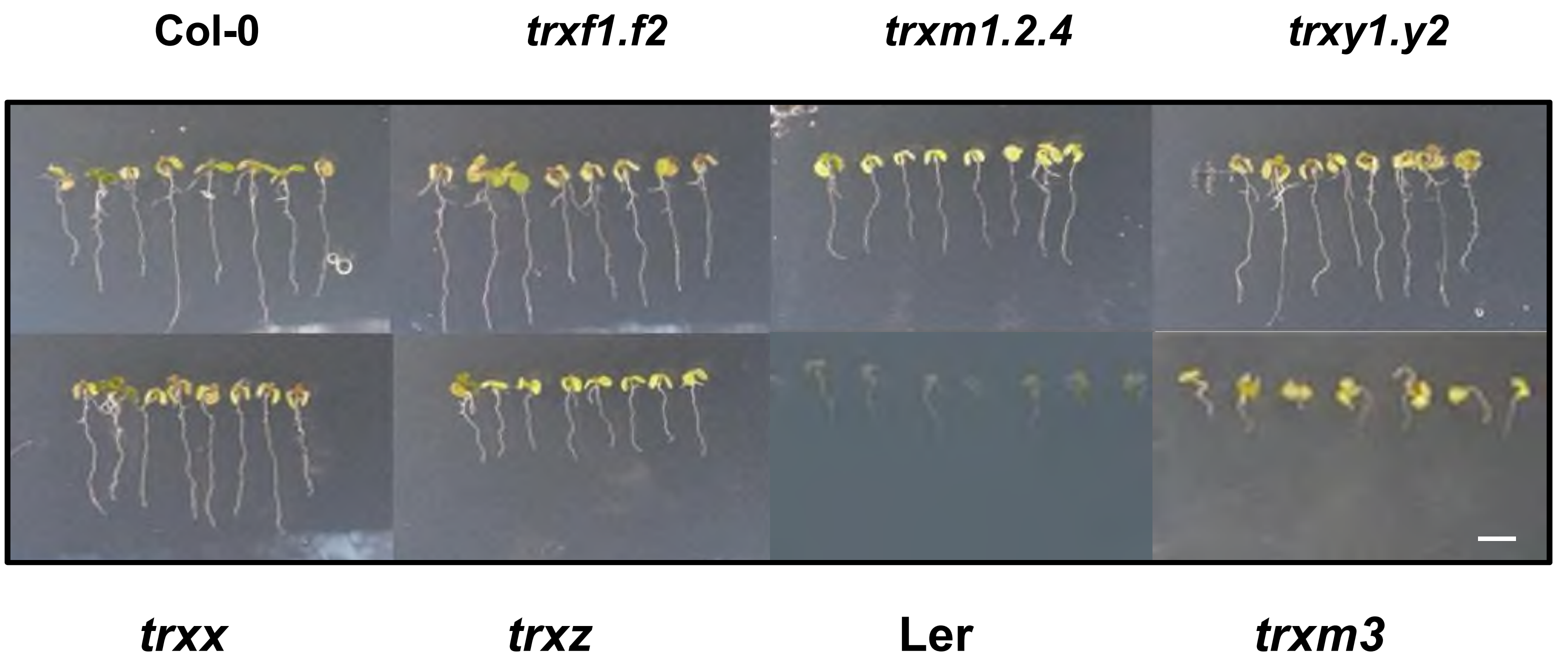

Sup. Figure 2. Root growth of *trx* mutants under high salinity.

Pictures of seedlings growing on ½ MS + 200 mM NaCl medium for 2 weeks, taken out from the culture box (darkened roots) for growth monitoring. Scale bar = 1cm.

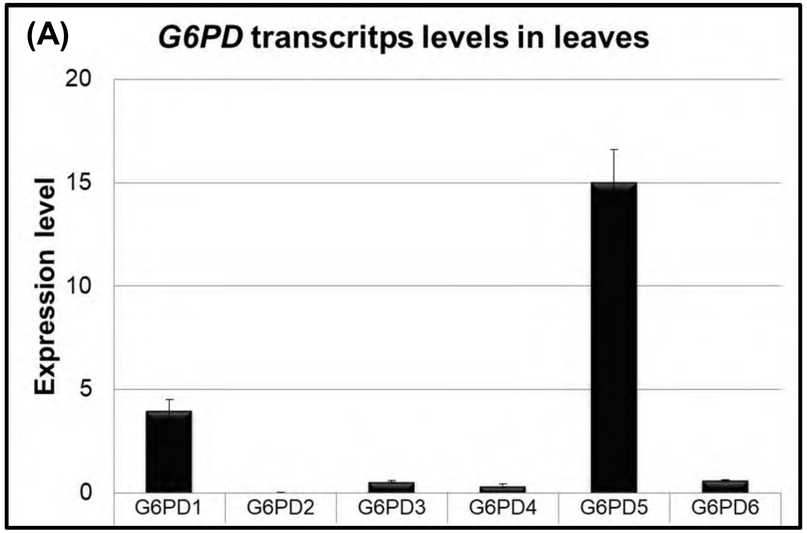

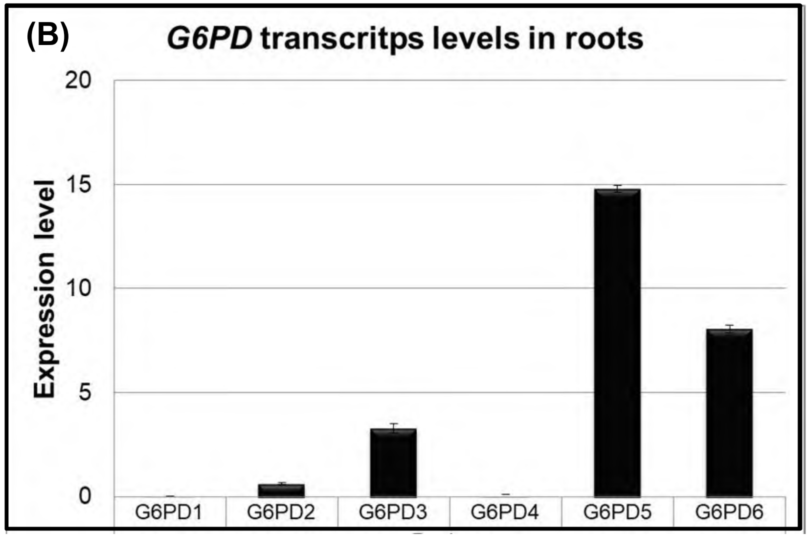

Sup. Figure 3. Expression levels of *G6PD* genes in Arabidopsis leaves and roots. mRNA were quantified by quantitative RT-PCR. Bars indicate standard deviation (n=6).

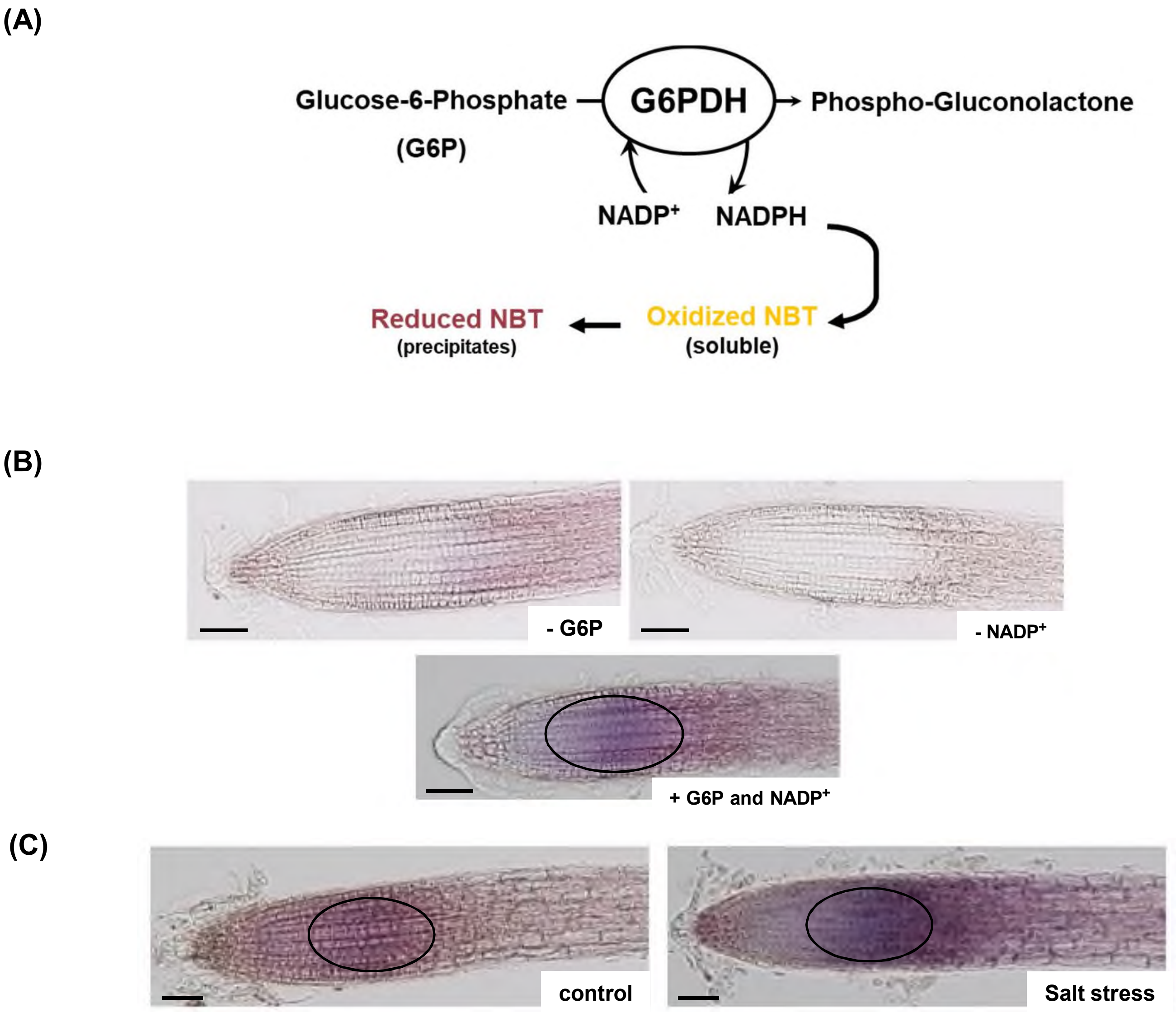

Sup. Figure 4. *In situ* G6PDH activity assay in Arabidopsis root tips.

(A) Reaction catalyzed using Nitro Blue Tetrazolium (NBT); (B) control tests of the *in situ* G6PDH activity assay; (C) effect of 100 mM NaCl on G6PDH activity**.** 8 day-old seedlings were analyzed. The area taken for densitometry analysis is depicted by a black oval. The ImageJ software was used for quantification. Scale bar = 50 µm. Representative images are shown.

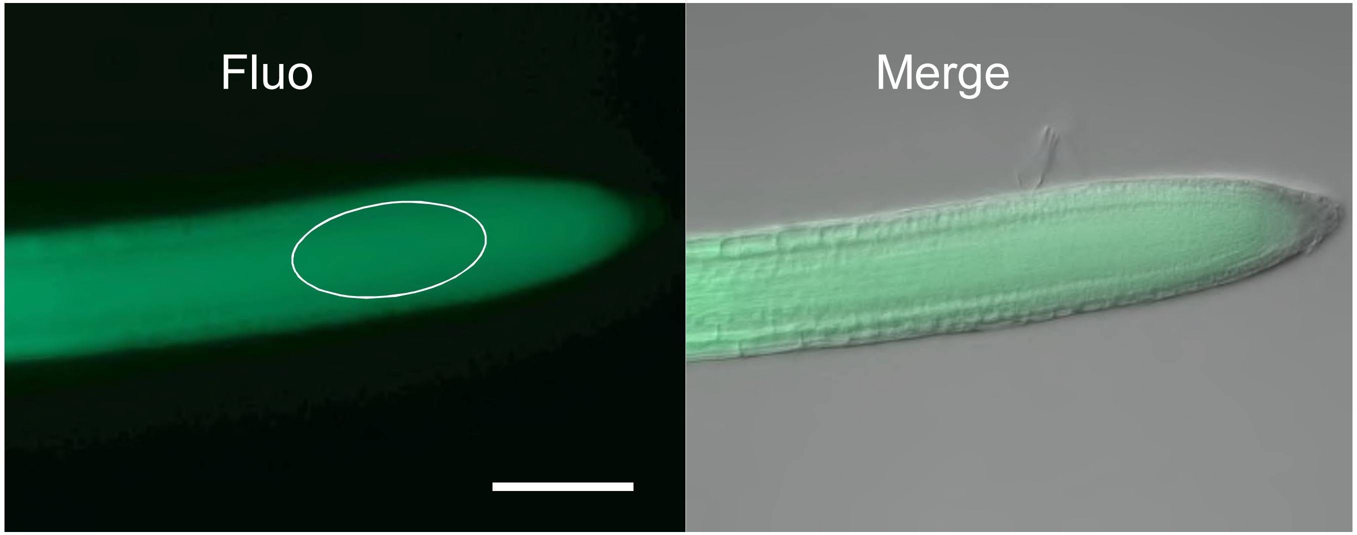

Sup. Figure 5. Area selected to quantify H2-DCFDA fluorescent signal in root tips.

Fluorescence was quantified in the root elongation zone (indicated by the white oval area) where *in situ* experiments have revealed localization of G6PDH activity. The ImageJ software was used for quantification. Bar = 10 µm. Exposure time 300 ms.

(A) Inhibition (reduction)

(B) Activation (oxidation)

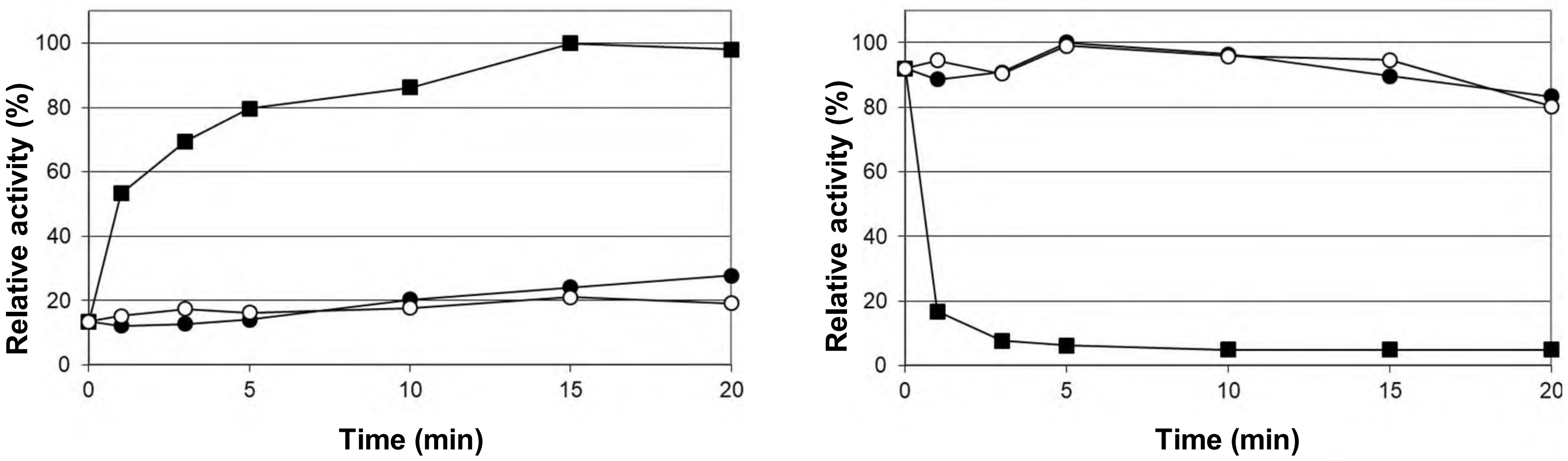

Sup. Figure 6. Redox regulation of G6PDH1 activity by TRX z.

Redox regulation of G6PDH1 activity was tested by incubating the enzyme with reduced (by 1 mM DTTred) or oxidized (by 10 mM DTTox) TRX (10 µM) prior to enzymatic activity measurements. DTT alone (○) had no effect at working concentrations, TRX f1 (■) and TRX z (●) efficiencies are compared.

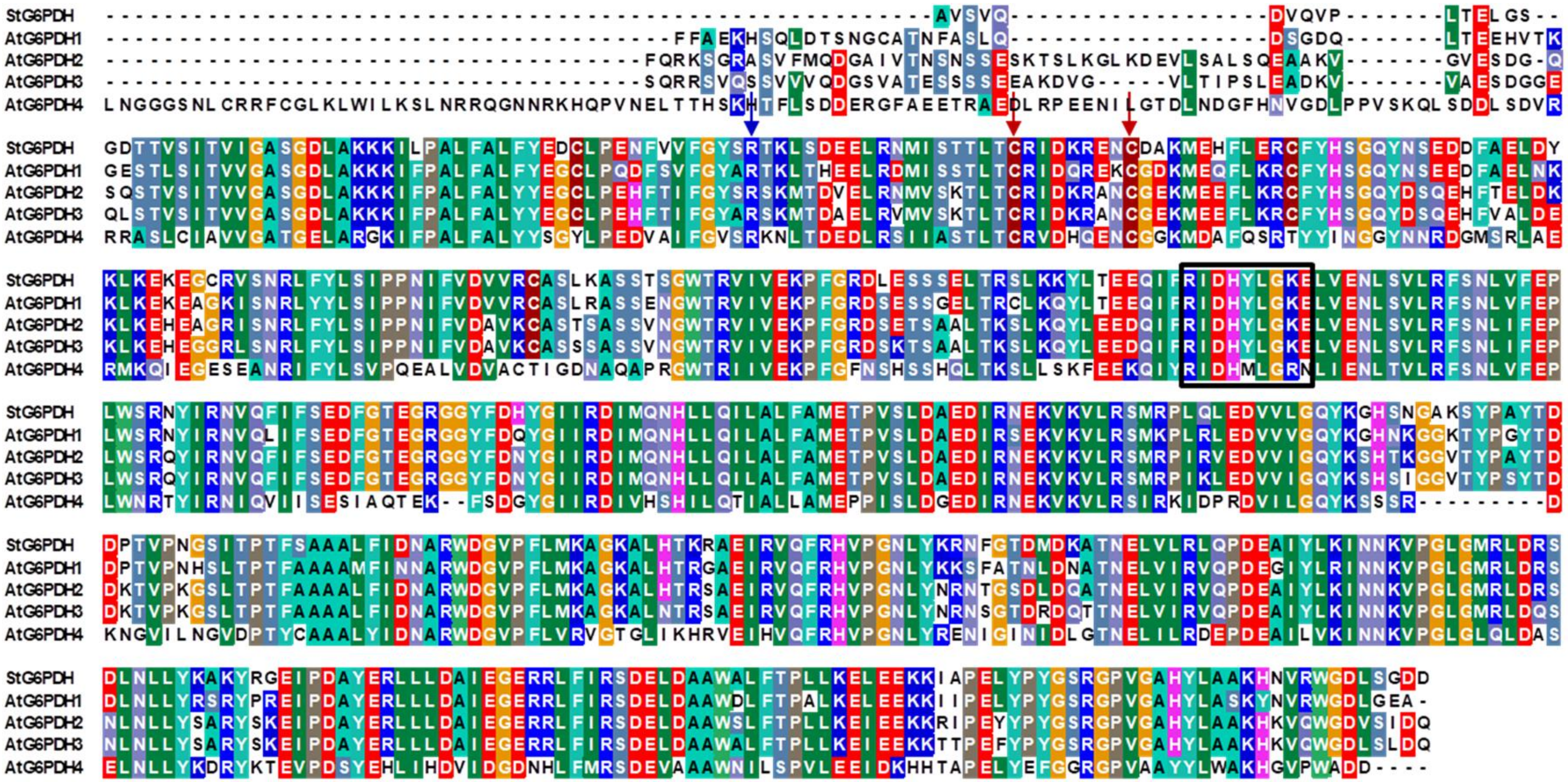

Sup. Figure 7. Amino acid sequence alignment of plastidial G6PDH isoforms.

The primary sequences of the 4 plastidial G6PDH isoforms from Arabidopsis (At) were aligned together with G6PDH from potato (St). Black box: active site signature / G6P binding site. Regulatory Cys (positions 149 and 157) and the Arg131 (conserved amino acids implicated in the TRX-dependent regulatory mechanism of plastidial G6PDH) are respectively indicated by burgundy and blue arrows. Numbering according to the AtG6PDH1 pre-mature form.

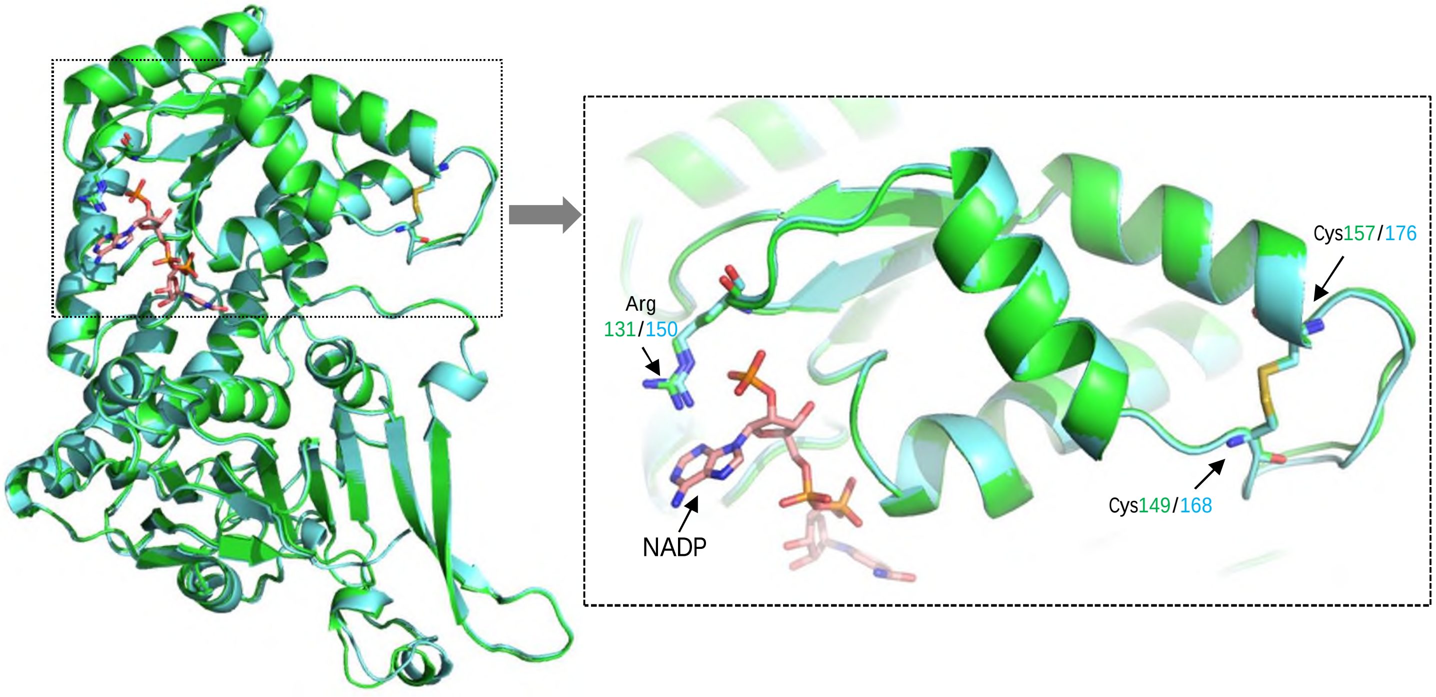

Sup. Figure 8. Comparison of Arabidopsis G6PDH1 and G6PDH2 3D structures.

Structural models of the G6PDH1 (UniProtKB_Q43727) and the G6PDH2 (UniProtKB_Q9FY99) monomeric subunit were downloaded from the AlphaFold repository (https://alphafold.ebi.ac.uk/). G6PDH1 (aa89-576) and G6PDH2 (aa108-596) were superimposed and shown in green and cyan, respectively. NADP cofactor was docked using CB-Dock2 (https://cadd.labshare.cn/cb-dock2/) and is shown as sticks, together with the regulatory cysteines bond in the TRX-dependent disulfide (in yellow) and the Arg131/150 of G6PDH1 / G6PDH2. The right panel shows a closeup view of the left panel squared region, corresponding to the active site region adjacent to the regulatory flexible loop. The Figure was prepared using PyMOL software (DeLano Scientific LLC).
